# The silent pharmacist: Harnessing the gut microbiome to improve therapy in hematologic malignancies

**DOI:** 10.1016/j.tranon.2026.102833

**Published:** 2026-05-25

**Authors:** Amr Ali Mohamed Abdelgawwad El-Sehrawy, Hamed Soleimani samarkhazan

**Affiliations:** a Internal Medicine, Diabetes, Endocrinology and Metabolism, Mansoura University, Mansoura, Egypt; b Student Research Committee, Department of Hematology and Blood Banking, School of Allied Medical Sciences, Shahid Beheshti University of Medical Sciences, Tehran, Iran

**Keywords:** Pharmacomicrobiomics, Hematologic malignancies, Gut microbiome, Immunotherapy, Personalized medicine

## Abstract

The gut microbiome, a complex ecosystem of microorganisms, is now recognized as a key determinant of drug efficacy and toxicity, giving rise to the field of pharmacomicrobiomics. This review decodes the profound influence of the gut microbiome on treatment outcomes for hematologic malignancies. We explore the tripartite mechanistic pathways through which gut microbes act: the direct enzymatic biotransformation of chemotherapeutic agents, the indirect immunomodulation of systemic and anti-tumor responses, and the preservation of mucosal barrier integrity to prevent devastating complications like graft-versus-host disease (GVHD). The manuscript details how the microbiome interacts with specific drug classes, from conventional chemotherapies like cyclophosphamide to cutting-edge immunotherapies like immune checkpoint inhibitors and CAR-T cells, shaping their clinical success. Furthermore, we discuss the translational potential of targeting this "silent pharmacist" through fecal microbiota transplantation, next-generation probiotics, and dietary interventions. Finally, we highlight the main translational opportunities, current limitations, and future clinical priorities for integrating microbiome science into hematology, paving the way for more personalized and improved cancer care.

## Introduction: the unseen variable in cancer therapy

The management of hematologic malignancies has advanced significantly, yet a major challenge persists: the extensive interpatient variability in treatment response and toxicity, even among patients with identical diagnoses receiving standardized protocols [1,2]. For decades, this variability was primarily attributed to host genetic factors, with pharmacogenomics identifying key polymorphisms (e.g., in TPMT affecting mercaptopurine metabolism) [3]. However, genetic factors alone explain only 20–95 % of pharmacokinetic and pharmacodynamic variability, leaving a substantial portion unexplained [2,4].

A paradigm shift has occurred with the recognition that the human body is a meta-organism, composed of human cells and a vast diversity of microbial inhabitants, collectively known as the microbiota [5,6]. This community, predominantly in the gastrointestinal tract, possesses a genetic repertoire (the microbiome) that far exceeds the human genome in scale and metabolic potential [5]. Far from being passive, the gut microbiome functions as a dynamic endocrine and metabolic organ, actively participating in nutrient extraction, vitamin synthesis, immune modulation, and the crucial biotransformation of xenobiotics [[5], [6], [7]]. It produces a diverse array of bioactive molecules (e.g., short-chain fatty acids, neurotransmitters) that enter systemic circulation and influence distal organs, thereby affecting host physiology and pharmacological responses [5,6].

This understanding has catalyzed the emergence of pharmacomicrobiomics, a field that seeks to characterize the intricate relationships between microbiome composition/function and drug disposition, efficacy, and toxicity [4,7,8]. It extends pharmacogenomics by incorporating the collective genetic contribution of the human-associated microbiota to drug response variability [7]. The fundamental premise is that variations in the microbiome, influenced by diet, age, geography, and medication history, can modify drug pharmacokinetics and pharmacodynamics through several mechanisms: direct microbial enzymatic transformation of drugs; alteration of host metabolic enzyme expression; modulation of intestinal permeability; and production of metabolites that interfere with drug transport or signaling [4,7,8]. Clinically, this is profound, as demonstrated by microbial β-glucuronidase reactivating irinotecan to its toxic form, causing severe diarrhea [7]. The gut microbiome influences drug response through a multitude of interconnected mechanisms, as summarized in Table 1.

**Table 1:** Mechanisms of microbiome influence on drug response.

Mechanism	Example	Clinical impact	Ref.
Direct biotransformation	Bacterial β-glucuronidase reactivates irinotecan (SN-38 G to SN-38)	Increased gastrointestinal toxicity, dose limitations	[7,13]
	Mycoplasma hyorhinis deaminates gemcitabine	Drug inactivation, reduced efficacy, potential contribution to resistance	[13]
Altered host metabolism	Microbial regulation of hepatic CYP450 enzymes (e.g., CYP3A4)	Modified drug bioavailability, metabolism, and clearance	[4,8]
Immunomodulation	Microbiota-dependent priming of dendritic and T cells (e.g., by SCFAs)	Altered response to immunotherapies (ICIs, CAR-T) and conventional chemotherapy	[17,18]
Barrier function modification	SCFA-mediated enhancement of intestinal tight junctions	Reduced bacterial translocation and systemic inflammation, lower GVHD risk	[4,18,19]
Production of bioactive metabolites	Butyrate (HDAC inhibitor) from Clostridium spp.	Modulation of gene expression, enhanced chemotherapy efficacy, anti-inflammatory effects	[13,18]

Recent clinical and translational studies reinforce the importance of these mechanisms in hematologic care. For example, FMT has shown promise for restoring microbial diversity and improving outcomes in steroid-refractory gastrointestinal graft-versus-host disease (aGVHD) [9]. Additionally, large analyses have linked pre-cellular therapy antibiotic exposure with worse outcomes and altered CAR-T cell responses, highlighting the clinical consequences of microbiome disruption in modern cellular therapies [10]. Finally, recent multi-omics work indicates that the gut microbiota conditions response to hypomethylating therapy (5-azacytidine) in myelodysplastic syndromes, suggesting that microbiome-drug interactions extend to standard hematology therapeutics [11].

In the context of hematologic malignancies, these pharmacomicrobiomic interactions are particularly significant for several reasons [8]. First, the extensive use of cytotoxic chemotherapeutic agents with narrow therapeutic indices means even modest alterations in drug metabolism can have profound consequences for efficacy and toxicity [2]. Second, the frequent administration of prolonged antibiotic prophylaxis during neutropenia dramatically alters microbiome composition and function, creating time-dependent fluctuations in drug-microbe interactions throughout treatment cycles [4]. Finally, the increasing implementation of immunotherapies (e.g., immune checkpoint inhibitors, CAR-T cells) introduces novel mechanisms by which microbiome-mediated immunomodulation can influence outcomes. Evidence shows the microbiome shapes systemic immune responses through pathways involving mucosal barrier integrity, antigen presentation, and T-cell differentiation, all of which could modify immunotherapy effectiveness [8]. This is supported by clinical observations linking antibiotic exposure to reduced immunotherapy efficacy in lymphoma patients, suggesting microbiome integrity is essential for an optimal treatment response [8]. The implications of pharmacomicrobiomics are particularly profound across the spectrum of hematologic malignancies. In acute leukemias, such as acute lymphoblastic leukemia (ALL) and acute myeloid leukemia (AML), the microbiome modulates the efficacy and mucosal toxicity of cornerstone agents like methotrexate and cytarabine [12,13]. For lymphomas, the response to immune checkpoint inhibitors (ICIs) is heavily influenced by commensal bacteria that prime anti-tumor immunity [14]. In multiple myeloma, the immunomodulatory effects of drugs like lenalidomide may be potentiated by a microbiome capable of supporting T-cell co-stimulation [14,15]. Furthermore, the success and toxicity profile of adoptive cell therapies, including CAR T-cells, are increasingly linked to pre-treatment microbial composition [14,16]. Therefore, this review focuses on how the gut microbiome modifies treatment response, toxicity, and biomarker development in hematologic malignancies.

## Mechanisms of the pharmacomicrobiome

The human gut microbial efficacy and toxicity via three closely linked pathways. First, direct microbial biotransformation, a diverse repertoire of microbial enzymes can activate prodrugs, inactivate active compounds, or generate toxic metabolites that alter systemic exposure. Second, immune modulation, microbial signals and metabolites (for example, short-chain fatty acids and tryptophan derivatives) recalibrate innate and adaptive immunity, thereby altering responses to cytotoxics and immuno mocosal barrier maintenance, third, microbiome-dependent preservation (or loss) of intestinal barrier integrity influences systemic inflammation, bacterial translocation and complications such as GVHD or sepsis. In hematologic malignancies, where dosing margins are narrow and immune status is frequently perturbed, these mechanisms have immediate clinical relevance for toxicity management and for optimizing the efficacy of cytotoxic, hypomethylating and cellular therapies [9,10,20].

### Direct biotransformation: microbial enzymes

The gut microbiota directly modifies drugs through a vast repertoire of microbial enzymes, performing transformations distinct from host metabolism. These microbial actions can activate prodrugs, inactivate active compounds, or generate toxic metabolites, fundamentally altering drug bioavailability and toxicity profiles [4].

Microbial nitroreductases are crucial for activating prodrugs like cyclophosphamide, a cornerstone of many hematologic malignancy regimens. Conversely, bacterial β-glucuronidases can reactivate glucuronidated metabolites, exacerbating toxicity. This is exemplified by irinotecan, where bacterial enzymes deconjugate the inactive metabolite SN-38 G back to the active and toxic SN-38 within the intestine, causing severe diarrhea and dose-limiting toxicities. Furthermore, the microbiota can inactivate drugs; for instance, *Mycoplasma hyorhinis* metabolizes gemcitabine via enzymatic deamination, reducing its therapeutic efficacy [13].

**Fig. 1:**
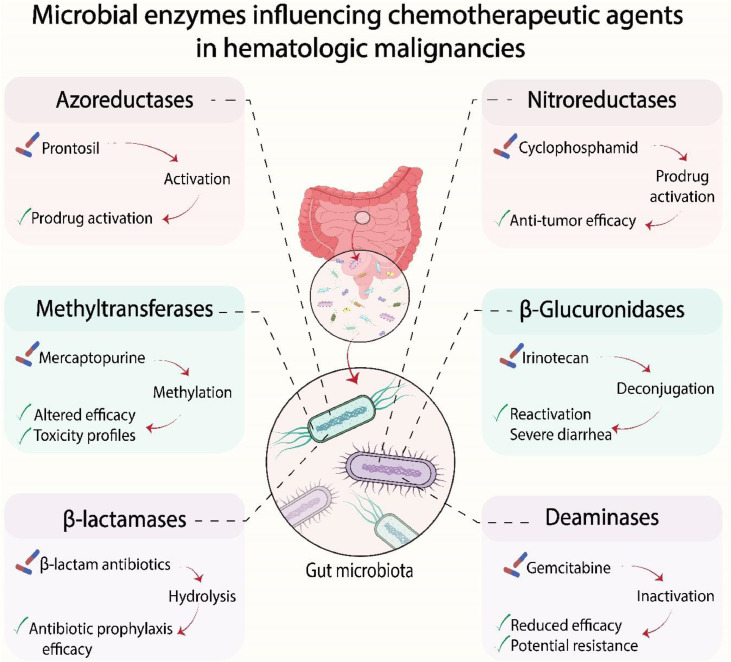
Several classes of microbial enzymes modulate the pharmacological activity of chemotherapeutic and supportive agents through activation, inactivation, or alteration of drug metabolism. These transformations can critically influence clinical outcomes by enhancing anti-tumor efficacy, precipitating dose-limiting toxicities, or contributing to treatment resistance.

Beyond direct drug modification, microbiota-derived metabolites such as SCFAs contribute to host immune regulation and epithelial homeostasis, and their clinical relevance is summarized in the sections below [[21], [22], [23]]. These complex enzymatic interactions, which can critically alter drug efficacy and toxicity, are summarized in Fig. 1.

### Indirect immunomodulation: priming host immunity

The gut microbiome profoundly shapes systemic immune responses, creating an immunologic context that can either support or hinder treatment efficacy in hematologic cancers through mechanisms including immune cell training, cytokine network regulation, and metabolite production [24]. The microbiome influences critical cellular populations like myeloid-derived suppressor cells (MDSCs). MDSCs differentiated with prostaglandin E2 (MDSC-PGE2) show enhanced immunosuppressive properties and can prevent gut barrier disruption and enrich microbial taxa with immunoregulatory properties, suggesting a reciprocal relationship between immune cells and microbes that influences treatment outcomes [24].

A key mechanism is the microbiome's regulation of T-cell differentiation. Specific commensals drive the differentiation of pro-inflammatory Th17 cells, while others, such as Bacteroides fragilis and Clostridium clusters, promote regulatory T cell (Treg) differentiation through SCFA production [17,21]. This balance between inflammatory and regulatory responses directly affects antitumor immunity.

Microbial metabolites are important mediators of this crosstalk and influence dendritic cells, macrophages, and T cells through several signaling pathways, including SCFA- and tryptophan-related mechanisms [21,25]. The microbiome also calibrates cytokine networks central to antitumor immunity. Specific taxa can induce Th1 cell responses or enhance production of cytokines like TNFα and IFNγ, establishing a systemic immunologic tone that may significantly influence responses to immunotherapies [17,25].

### Guardian of the gate: microbiome and mucosal barrier integrity

The gut microbiome is a critical guardian of intestinal barrier integrity, a function highly relevant in hematologic malignancies where chemotherapy-induced mucositis and graft-versus-host disease (GVHD) are major complications [4,26,27]. An intact microbiome prevents bacterial translocation by maintaining epithelial barrier integrity. Specific taxa like Akkermansia muciniphila and Faecalibacterium prausnitzii strengthen barrier function by enhancing mucus production and tight junction protein expression [4]. Conversely, dysbiosis with expansion of pathobionts like *E. coli* increases intestinal permeability, facilitating the translocation of microbial products (e.g., LPS) into circulation, which can trigger inflammatory responses and exacerbate complications like GVHD and sepsis [4]. In allogeneic hematopoietic stem cell transplantation (allo-HSCT), pre-transplant microbiome composition is a powerful predictor of GVHD risk and survival. Loss of diversity and expansion of Enterococcus and *E. coli* correlate with increased GVHD incidence and severity, while butyrate-producing bacteria like Faecalibacterium and Roseburia demonstrate protective effects by enhancing epithelial integrity and promoting Treg [19,21]. Barrier protection is mediated in part by microbial metabolites, but the clinically important point is that preserved microbiome diversity supports mucosal integrity and may reduce translocation, GVHD, and infectious risk [21,25].

This guardian role has profound implications. Preserving microbiome diversity supports mucosal homeostasis, potentially reducing complications and enabling treatment intensification. Conversely, microbiome disruption compromises barrier function, contributing to systemic inflammation. Therefore, microbiome-targeted interventions represent a promising strategy for improving outcomes, especially in the context of intensive chemotherapy and transplantation [4,26]. Beyond barrier integrity, gut-derived signals may also influence hematopoiesis and the bone marrow niche, creating a mechanistic bridge to leukemogenesis and treatment response.

### The gut-immune-BM axis: microbial metabolites and hematopoiesis

Beyond local intestinal and systemic immunomodulatory effects, the gut microbiome may also influence the bone marrow microenvironment through microbial metabolites, translocated bacterial components, and immune signals. This gut-immune-bone marrow axis is biologically plausible and may shape hematopoiesis, stromal-cell signaling, and the leukemic niche [28,29].

Microbial metabolites, particularly short-chain fatty acids (SCFAs) like butyrate and propionate, can enter systemic circulation and reach the BM. There, they influence hematopoietic stem and progenitor cell (HSPC) function. Butyrate, as a histone deacetylase inhibitor, may alter the epigenetic landscape of HSPCs, potentially affecting their self-renewal, differentiation, and susceptibility to malignant transformation [30,31]. SCFAs also signal through G-protein-coupled receptors (GPCRs) such as GPR41 and GPR43 on BM stromal cells and immune cells, modulating the production of hematopoietic cytokines (e.g., GM-CSF, G-CSF) and creating a BM microenvironment that can either suppress or promote leukemogenesis [29,32]. Furthermore, microbiome-derived signals are integral to trained immunity, a functional reprogramming of innate immune cells that can occur in the BM. For instance, microbial components like lipopolysaccharide (LPS) can prime BM-derived myeloid cells, altering their long-term inflammatory tone and response to subsequent challenges, which may impact the initiation or progression of myelodysplastic syndromes (MDS) and AML [[33], [34], [35], [36]]. This axis may also have therapeutic implications, because the efficacy of certain chemotherapies and hypomethylating agents could be influenced by microbiome effects on the marrow niche and immune surveillance [12,[37], [38], [39]]. Dysbiosis-induced disruption of this axis may contribute to BM suppression, increased infection risk during neutropenia, and altered regenerative capacity post-transplantation, underscoring the microbiome's role as a systemic regulator of the primary site of hematologic disease.

## Microbiome-drug interactions in hematologic malignancies

This section details the complex interplay between the gut microbiome and major therapeutic classes used in hematologic malignancies, exploring how microbial communities influence drug efficacy, toxicity, and resistance.

### Conventional chemotherapies and targeted agents

The efficacy and toxicity of conventional chemotherapies are profoundly shaped by the gut microbiome through enzymatic biotransformation, immunomodulation, and maintenance of intestinal barrier integrity.

#### Cyclophosphamide and Ifosfamide

The antitumor efficacy of these alkylating agents is significantly enhanced by specific gut bacteria through immunomodulatory mechanisms. Enterococcus hirae translocates to secondary lymphoid organs, stimulating pathogenic Th17 and memory Th1 immune responses crucial for the drug's effect [14,15]. Concurrently, Barnesiella intestinihominis facilitates the recruitment of interferon-γ-producing γδ T cells and cytotoxic CD8+ T cells into the tumor microenvironment [15]. The microbiome also influences the intestinal inflammation and mucosal damage caused by cyclophosphamide, impacting treatment tolerance.

#### Methotrexate

This antifolate agent exhibits a dual relationship with the microbiome. Pro-inflammatory microbiota exacerbate methotrexate-induced intestinal damage and mucositis, while anti-inflammatory species like Lactobacillus and Bifidobacterium demonstrate protective effects by preserving mucosal integrity and reducing inflammation [12,14,40]. Methotrexate itself induces dysbiosis, characterized by reduced diversity and depletion of SCFA-producing bacteria, which may further amplify toxicity and impair immune function [14].

#### 5-Azacytidine/decitabine (hypomethylating agents)

Emerging evidence suggests the gut microbiome modulates the efficacy of HMAs, cornerstone therapies for myelodysplastic syndromes and AML. The microbiome may regulate the drugs' effects through immune modulation and the production of metabolites like SCFA [14,15,41]. Preliminary data indicates that enriched populations of SCFA-producing bacteria are associated with improved outcomes, potentially by enhancing dendritic cell function and T-cell mediated immunity [14,16]. The microbiome may also influence the inflammatory tumor microenvironment, affecting responses to HMA combination therapies.

#### Immunomodulatory imide drugs (IMiDs - Lenalidomide, Pomalidomide)

The efficacy of IMiDs in multiple myeloma is largely dependent on T-cell co-stimulation, an effect potentially potentiated by the gut microbiome. Specific bacteria like Faecalibacterium prausnitzii and Akkermansia muciniphila may enhance IMiD-induced T-cell activation by regulating dendritic cell function [14,15,42]. Furthermore, microbiota-derived metabolites, including SCFAs and tryptophan derivatives, may influence IMiD efficacy by modulating T-cell polarization and function [14,16].

### The microbiome-immunotherapy axis

The microbiome is a crucial determinant of success for immunotherapies, modulating host immune responses both locally and systemically.

#### Immune checkpoint inhibitors (ICIs - Anti-PD-1/PD-L1, CTLA-4)

Although most evidence comes from solid tumors, lessons are highly relevant to lymphomas like cHL and PMBCL. Specific bacterial consortia are consistently associated with improved ICI responses. Akkermansia muciniphila, Bifidobacterium spp., and Faecalibacterium prausnitzii enhance dendritic cell antigen presentation and CD8+ T-cell tumor infiltration [14,15,43]. These bacteria produce metabolites (SCFAs, inosine) that stimulate T-cell function [14,16]. Conversely, dysbiosis with reduced diversity and depletion of these taxa is linked to primary resistance and increased immune-related adverse events [14].

#### Chimeric antigen receptor T-cell (CAR-T) therapy

The microbiome appears to influence the three major challenges of CAR-T therapy: Cytokine Release Syndrome (CRS), Immune Effector Cell-Associated Neurotoxicity Syndrome (ICANS), and CAR-T cell persistence. Patients with microbiome signatures enriched in Faecalibacterium and Akkermansia demonstrate reduced incidence and severity of CRS/ICANS, likely through regulation of inflammatory cytokines and maintenance of gut integrity. Microbial metabolites like SCFAs enhance memory T-cell generation, potentially promoting long-term CAR-T persistence [14,16,44]. Critically, broad-spectrum antibiotic use prior to CAR-T infusion causes dysbiosis and is associated with reduced CAR-T expansion and poor clinical outcomes [12,14,40].

### The janus face: microbiome and treatment-related toxicity

The microbiome plays a dual role, both protecting against and contributing to adverse effects.

#### GVHD

In allogeneic HSCT, microbiome dysbiosis is a key driver of GVHD. Loss of diversity and depletion of commensals like Blautia and Faecalibacterium are strongly associated with increased GVHD incidence and severity [14]. These protective bacteria produce SCFAs like butyrate, which serve as energy for colonic epithelial cells and have anti-inflammatory properties, enhancing regulatory T-cell function and promoting mucosal healing [14,16]. Pre-transplant dysbiosis can predict subsequent GVHD, offering a window for preemptive intervention.

#### CRS and ICANS

Dysbiosis may exacerbate these toxicities through intestinal barrier disruption and systemic inflammation. Bacterial translocation across a compromised gut barrier activates innate immune cells, leading to excessive production of IL-6, IL-1, and TNF-α that drive CRS [45]. The microbiome may influence ICANS by regulating blood-brain barrier integrity; protective metabolites like SCFAs help maintain this barrier and reduce neuroinflammation [16,46]. Patients with microbiome disruption prior to CAR-T therapy experience more severe toxicities [47].

#### Infectious complications

The microbiome provides colonization resistance against pathogens during neutropenia. However, necessary prophylactic antibiotics exacerbate dysbiosis, leading to a loss of diversity and expansion of antibiotic-resistant pathogens like Enterococcus spp. and *E. coli*, which can translocate and cause bloodstream infections [12,14,40]. Specific commensals, such as Blautia producta and Clostridium scindens, inhibit colonization by vancomycin-resistant enterococcus (VRE) and C. difficile, respectively [14]. Microbiome-derived metabolites like SCFAs also stimulate neutrophil function, enhancing antimicrobial defense [14,15]. This highlights the need for targeted antimicrobial approaches to preserve protective microbiota. The relationship between microbiome disruption and treatment-related toxicities is complex, with specific dysbiotic patterns linked to distinct complications through proposed mechanisms summarized in Table 2.

**Table 2:** Microbiome features associated with treatment-related toxicities and proposed interventions.

Toxicity	Microbiome features	Proposed mechanisms	Potential interventions	Ref.
GI GVHD	Loss of diversity; depletion of *Blautia, Faecalibacterium*	Impaired SCFA production, intestinal barrier disruption, increased inflammation	FMT, SCFA administration	[19,48,49]
CRS (CAR-T therapy)	Reduced diversity; enterococcal dominance	Bacterial translocation, innate immune activation, excessive cytokine (e.g., IL-6) production	Prebiotic fibers, targeted probiotics	[16,45,46]
ICANS (CAR-T therapy)	Depletion of SCFA-producers	Blood-brain barrier disruption, neuroinflammation	Microbial metabolite supplementation	[16,46,50]
Chemotherapy-induced mucositis	Loss of mucosal-associated commensals (e.g., Lactobacilli)	Epithelial damage, impaired repair, increased local inflammation	Probiotics (e.g., *L. rhamnosus* GG)	[51,52]
Bloodstream infections	Loss of diversity; expansion of *Enterococcus, E. coli*	Loss of colonization resistance, translocation of pathogens across compromised barrier	Targeted antimicrobial prophylaxis	[40,51,52]

## Methodological toolkit: a translational framework for microbiome studies

Deciphering host–microbiome–drug interactions in hematologic malignancies requires a focused translational framework rather than an overly broad methodological inventory. In practice, the most informative studies combine microbiome profiling with functional readouts and clinical correlation. Sequencing approaches such as 16S rRNA profiling or shotgun metagenomics can define microbial composition and potential function, while metatranscriptomics and metabolomics help capture microbial activity and metabolite output relevant to treatment response and toxicity [[53], [54], [55], [56], [57], [58], [59]].

Causal validation should rely on targeted experimental systems, including germ-free or gnotobiotic mouse models and fecal microbiota transplantation experiments, to test whether specific microbial patterns directly modify drug effects or immune outcomes [[60], [61], [62]]. For clinical translation, multimodal data integration and longitudinal sampling are most useful when they are tightly linked to clinically meaningful endpoints such as response, toxicity, infection risk, or GVHD. This streamlined approach keeps the focus on translational relevance while avoiding unnecessary technical detail.

## Translational roadmap: microbiome-targeted therapies

The growing understanding of the pharmacomicrobiome is driving the development of innovative strategies to modulate microbial communities and improve clinical outcomes in hematologic malignancies. This section outlines the roadmap for harnessing the microbiome as a predictive biomarker, therapeutically targeting it, and designing robust clinical trials to validate these approaches [63,64].

### Microbiome as a biomarker: pre-treatment microbial signatures

The gut microbiome serves as a powerful predictive biomarker for treatment response and toxicity. Advances in sequencing and bioinformatics have enabled the identification of specific microbial signatures that correlate with clinical outcomes, allowing for risk stratification and treatment personalization [65].

Pre-treatment microbial diversity and composition are strongly predictive of outcomes in hematopoietic stem cell transplantation (HSCT). Higher gut microbial diversity pre-transplant is associated with significantly reduced complications, including lower rates of GVHD and improved overall survival [63,66]. Specifically, the abundance of butyrate-producing bacteria like Faecalibacterium prausnitzii and Roseburia species is linked to reduced GVHD severity and mortality, while dominance of Enterococcus species correlates with increased GVHD incidence and worse survival [48,66,67].

**Fig. 2:**
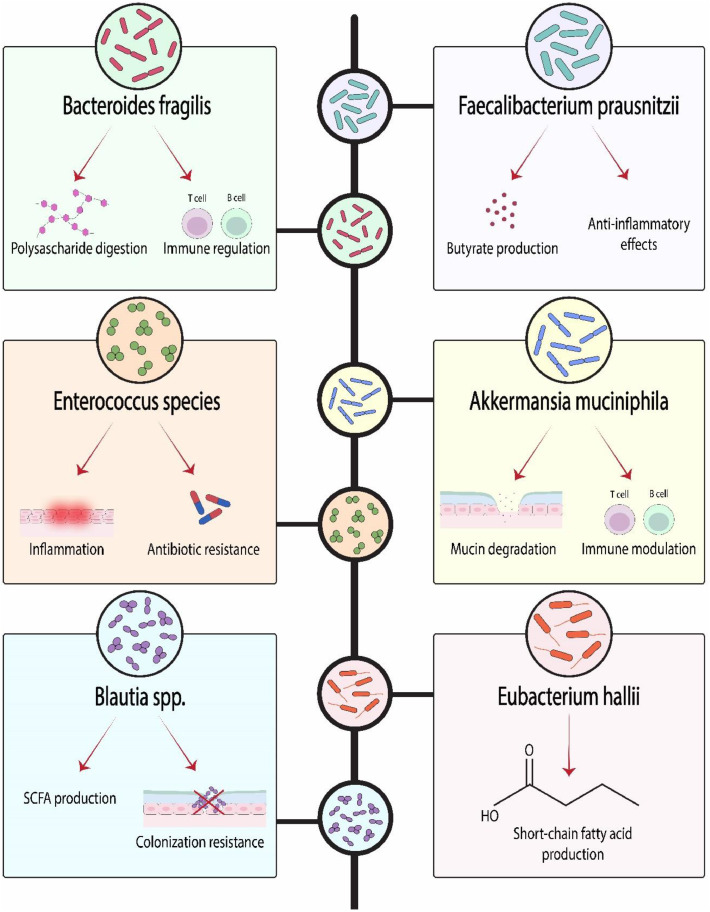
Key gut microbial taxa with defined metabolic and immunomodulatory functions are shown. These microbes contribute to processes such as short-chain fatty acid production, mucin degradation, inflammation control, and immune regulation, underscoring their potential relevance in the context of hematologic.

Beyond HSCT, microbiome biomarkers show promise in predicting responses to chemotherapy and immunotherapy. For instance, specific patterns involving Akkermansia muciniphila and Bacteroides species are associated with improved responses to immune checkpoint inhibitors in lymphoma patients [63,67]. The development of standardized biomarkers faces challenges like technical variability and geographical differences, but large microbiome biobanks and ML approaches are helping to develop robust predictive models that integrate microbial features with clinical variables [65,[68], [69], [70]]. This predictive potential is increasingly validated in prospective clinical settings. For instance, in patients undergoing allogeneic HSCT, prospective profiling has confirmed that low microbial diversity and high abundance of *Enterococcus* prior to transplant are independent risk factors for lethal GVHD and inferior overall survival [71]. Similarly, in trials of immune checkpoint inhibitors for lymphoma, baseline enrichment of Akkermansia muciniphila has been prospectively associated with significantly longer progression-free survival, providing a potential biomarker for patient stratification [72]. The predictive power of the gut microbiome is increasingly recognized, with specific microbial signatures correlating strongly with clinical outcomes, as presented in Table 3. The key functional roles of these predictive microbial taxa are illustrated in Fig. 2.

**Table 3:** Predictive microbial biomarkers in hematologic malignancies and their clinical correlations.

Microbial taxon	Associated function	Clinical correlation	Ref.
*Faecalibacterium prausnitzii*	Butyrate production, anti-inflammatory effects	Reduced GVHD severity, improved overall survival post-HSCT	[48,67]
*Akkermansia muciniphila*	Mucin degradation, immune modulation	Improved response to immunotherapy (ICIs)	[63,67]
*Eubacterium hallii*	Short-chain fatty acid production	Reduced transplantation-related complications	[66,67]
*Blautia* spp.	SCFA production, colonization resistance	Protection against GVHD and VRE colonization	[49,51]
*Enterococcus* species	Inflammation, antibiotic resistance	Increased GVHD incidence, worse survival	[48,66]
*Bacteroides fragilis*	Polysaccharide digestion, immune regulation	Associated with improved chemotherapy response	[63,67]

### Therapeutic interventions: from microbial modulation to engineered therapeutics

Therapeutic manipulation of the microbiome represents a promising approach to improving outcomes in hematologic malignancies. Current strategies range from broad ecological restoration approaches like FMT to highly targeted engineered probiotics designed for specific therapeutic functions. Each approach offers distinct mechanisms of action, advantages, and challenges for clinical implementation. A diverse arsenal of microbiome-targeted interventions is currently under investigation, each with distinct mechanisms and developmental stages, as outlined in Table 4.

**Table 4:** Microbiome-targeted therapeutic strategies and their development status.

Intervention type	Examples	Mechanism of action	Clinical development stage	Ref.
FMT	Healthy donor stool	Restore microbial diversity and function, reduce pathogens	Phase II/III trials for GVHD	[48,49]
Next-generation probiotics (NGPs)	*A. muciniphila, F. prausnitzii*	Immunomodulation, barrier enhancement, SCFA production	Phase I/II trials	[67,68]
Dietary interventions	High-fiber, Mediterranean diets	Modulate microbial composition and metabolite production	Observational and small trials	[68,73,74]
Prebiotics	Inulin, Galacto-oligosaccharides	Selective stimulation of beneficial bacteria (e.g., Bifidobacteria)	Preclinical and early clinical	[67]
Engineered biotherapeutics	EcN producing therapeutic enzymes	Targeted drug delivery, metabolite production, toxin degradation	Preclinical development	[64]
Defined microbial consortia	Bacterial "cocktails"	Enhanced colonization, synergistic metabolic activities	Preclinical development	[64,67]
Post-therapy recovery support	FMT, SCFA supplements	Promote hematopoietic niche integrity, support balanced immune reconstitution (e.g., Treg expansion)	Early-phase exploratory trials	[75]

#### Prebiotics and probiotics

Prebiotics (dietary components that promote beneficial microbes) and probiotics (live beneficial microorganisms) constitute the most accessible approach to microbiome modulation. Current evidence supports the use of specific probiotic strains, particularly Lactobacillus and Bifidobacterium species, for reducing complications such as mucositis and diarrhea in patients undergoing chemotherapy or HSCT [67,76]. However, the efficacy of conventional probiotics in hematologic malignancies remains limited by strain-specific effects, inadequate colonization resistance, and potential risks in immunocompromised patients [63,67].

Next-generation probiotics (NGPs), including *Akkermansia muciniphila, Faecalibacterium prausnitzii*, and *Bacteroides* species, offer enhanced therapeutic potential compared to traditional probiotics due to their origin from human gut microbiota and their key roles in maintaining intestinal homeostasis [67]. These NGPs demonstrate enhanced ability to produce immunomodulatory metabolites such as SCFAs, maintain intestinal barrier integrity, and suppress pathogenic organisms [64,67]. However, their implementation faces technical challenges related to cultivation requirements, storage stability, and safety validation in immunocompromised hosts [[67], [68], [69]]. A pivotal randomized trial in allogeneic HSCT patients demonstrated that a prophylactic probiotic (*Lactobacillus plantarum*) significantly reduced the incidence of severe acute GVHD and improved survival, providing high-level evidence for strain-specific efficacy [77,78]. This underscores the need for rigorous, controlled trials to establish both the long-term safety, particularly regarding infection risk from bacteremia, and the durable efficacy of probiotic interventions in this vulnerable population.

#### Dietary modulation

Dietary intervention represents a powerful yet underutilized approach to shaping the gut microbiome. Specific dietary patterns significantly influence microbial composition and function, potentially affecting treatment outcomes in hematologic malignancies [68,69]. For instance, high-fiber diets promote the growth of butyrate-producing bacteria that strengthen gut barrier function and reduce inflammation, while low-carbohydrate diets may suppress the expansion of inflammatory pathobionts [48,67]. Despite the compelling rationale for dietary interventions, clinical implementation faces challenges including poor adherence in ill patients, variable individual responses to dietary changes, and limited high-quality evidence from randomized trials [68,69]. Ongoing research is focusing on developing personalized nutritional approaches based on individual microbiome composition and metabolic needs, which may enhance the efficacy and consistency of dietary interventions in hematologic patients [[68], [69], [70]].

#### FMT

FMT has emerged as a promising strategy for restoring a favorable microbial ecosystem after antibiotic disruption, potentially improving responses to immunotherapy and mitigating GVHD. The rationale for FMT in hematologic malignancies is particularly strong for patients with steroid-resistant GVHD, where restoration of microbial diversity may break the cycle of inflammation and tissue damage [48,66]. Recent clinical studies demonstrate that FMT can induce clinical responses in 60–80 % of patients with steroid-resistant GI GVHD, with complete response rates exceeding 50 % in some cohorts [66]. For instance, a phase II trial (NCT02733744) reported that single-dose FMT led to a 70 % overall response rate in steroid-refractory GI-GVHD, with durable responses observed [79]. Another trial demonstrated the efficacy of FMT in decolonizing multidrug-resistant organisms in patients with hematologic malignancies, highlighting its multifaceted utility [80]. The mechanisms underlying these benefits likely involve restoration of protective taxa, reduction of pathogenic organisms, and reestablishment of microbial metabolic functions that promote intestinal homeostasis and immune regulation. However, safety concerns remain particularly relevant in immunocompromised patients, including the risk of pathogen transmission and the potential for immune-related adverse events [48,66]. These concerns were highlighted by reports of extended-spectrum beta-lactamase (ESBL)-producing *E. coli* bacteremia and fatalities following FMT in immunocompromised patients, underscoring the non-negotiable need for rigorous, multi-step donor screening [81]. Despite risks, clinical data are compelling. A phase II trial (NCT03720392) in steroid-refractory GI-GVHD reported an overall response rate of 77 % at week 4 post-FMT, with complete responses in 33 % of patients, correlating with the restoration of butyrate producers like Faecalibacterium [[82], [83], [84], [85], [86]]. Ongoing trials are exploring FMT earlier in the treatment course, such as post-antibiotic prophylaxis or in combination with CAR-T therapy to mitigate toxicity, which will better define its optimal timing and safety profile [[87], [88], [89]]. Several ongoing clinical trials are evaluating FMT in hematologic malignancies, including studies combining FMT with immunotherapy to enhance antitumor responses, trials using FMT to decolonize multidrug-resistant organisms, and investigations of FMT for preventing GVHD when administered early after transplantation [48,66]. The development of standardized screening protocols, optimized delivery methods, and defined microbial communities may address current limitations and expand the therapeutic applications of FMT in hematology [66,68,69]. Safety, donor-screening, and regulatory considerations are discussed in Section 6.3.

#### Next-generation biotherapeutics

Beyond conventional microbiome-based therapies, next-generation biotherapeutics include defined bacterial consortia ("bacterial cocktails") and engineered probiotics designed for targeted drug delivery or metabolite production [64]. These approaches offer enhanced precision, consistency, and safety compared to conventional probiotics or FMT. Synthetic biology has enabled the engineering of probiotic bacteria to perform therapeutic functions beyond their native capabilities. For example, *E. coli* Nissle 1917 has been engineered to produce phenylalanine ammonia-lyase for treatment of phenylketonuria, secrete anti-inflammatory cytokines, or degrade toxic metabolites. Similarly, Bacteroides species have been modified to inactivate oncogenic metabolites or deliver nanobodies targeting tumor antigens [64,[90], [91], [92]].

The development of these engineered therapeutics requires careful consideration of genetic circuit design, selection of appropriate microbial chassis, and addressing patient-specific factors such as dosage, delivery method, and safety. Advanced synthetic biology tools including CRISPR-Cas systems, tailored promoters, and genetic circuits that respond to environmental signals have accelerated the engineering of gut commensals for therapeutic applications [64,93]. The translational potential of this approach is being actively tested. A first-in-human trial of an engineered strain of *E. coli* Nissle 1917, modified to consume ammonia, demonstrated safety and proof-of-concept in patients with hepatic encephalopathy, providing a precedent for similar applications in oncology [94,95]. In hematology, early-phase trials are planned for engineered probiotics designed to degrade immunotoxic metabolites in the gut post-HSCT or to deliver localized immune adjuvants to enhance CAR-T function, representing the frontier of precision microbiome engineering.

An additional translational implication of microbiome modulation is post-therapy recovery. By supporting epithelial integrity and SCFA production, microbiome restoration may contribute to hematopoietic recovery and immune reconstitution after HSCT or chemotherapy, although this remains an early and still largely inferential area that requires prospective validation [75,96].

### Clinical trial design: considerations for interventional trials

The translation of microbiome science into clinical practice requires well-designed interventional trials that address the unique challenges of microbiome-targeted therapies. Key considerations include patient stratification, timing of intervention, endpoint selection, and standardization of methodologies across study sites [[68], [69], [70]]. Attention to these design elements is critical for generating robust, reproducible evidence that can support regulatory approval and clinical adoption.

Patient stratification based on baseline microbiome features represents a crucial approach for enriching trials with patients most likely to benefit from microbiome-targeted interventions [[68], [69], [70]]. For example, patients with low microbial diversity or specific dysbiosis patterns may be more likely to respond to FMT or probiotic administration than those with preserved microbiome structure [66,70]. The integration of microbiome biomarkers with clinical variables may enable the identification of patient subgroups that derive maximal benefit from specific interventions, ultimately supporting personalized microbiome medicine approaches [65,68,69].

The timing of microbiome interventions represents another critical consideration in trial design. Interventions may be most effective when administered during specific therapeutic windows, such as before conditioning chemotherapy, during antibiotic administration, or early after HSCT [48,63]. The dynamic nature of the microbiome during cancer therapy further complicates timing decisions, as the susceptibility to modulation may vary throughout treatment [63,70]. Adaptive trial designs that allow for intervention timing based on individual microbiome trajectories may optimize therapeutic efficacy but present logistical challenges for implementation [70].

Endpoint selection for microbiome clinical trials requires careful consideration of clinical relevance, regulatory acceptance, and practical feasibility. While composite endpoints incorporating microbiome features are scientifically appealing, regulatory agencies typically require clinically meaningful endpoints such as overall survival, progression-free survival, or validated patient-reported outcomes [70,97]. For microbiome trials specifically, appropriate endpoints may include reduction in GVHD severity, decreased antibiotic use, lower infection rates, improved treatment response, or reduced toxicity [19,48,70].

The standardization of methodologies across study sites is essential for generating reliable, reproducible microbiome data in multicenter trials. Variability in sample collection, processing, sequencing, and bioinformatic analysis can introduce substantial noise that obscures true treatment effects [65,68,69]. Recent initiatives such as the Human Microbiome Action Project, the Strengthening the Organization and Reporting of Microbiome Studies (STORMS) checklist, and the Microbiome Quality Control Project provide frameworks for standardizing microbiome research [68,69]. Implementation of these standards in clinical trials will enhance data quality and comparability across studies. The statistical design of microbiome trials must account for multiple testing, high-dimensional data, and potential confounders such as diet and medications [70]. False-positive error control is particularly important in microbiome studies due to the large number of taxonomic features analyzed simultaneously [70]. Adaptive designs, Bayesian methods, and ML approaches offer promising strategies for analyzing complex microbiome data while maintaining statistical rigor [65,70].

Finally, the safety monitoring of microbiome-targeted therapies requires special consideration due to the potential for unexpected ecological effects, horizontal gene transfer in engineered microbes, and long-term consequences of microbial manipulation [64,66]. Extended follow-up periods and careful documentation of adverse events are essential for fully characterizing the risk-benefit profile of these novel interventions [66,70].

## Challenges and future perspectives

### Technical and methodological challenges

The field faces significant technical hurdles, primarily the difficulty of analyzing low microbial biomass in blood and bone marrow samples, which complicates distinguishing true microbial signals from contamination. While advances like microbial single-cell sequencing (e.g., smRandom-seq2) are emerging, they require further refinement [98]. Another major challenge is the integration of multi-omics data (genomics, transcriptomics, metabolomics) to move beyond correlative observations and establish causal mechanisms [52,[99], [100], [101]]. Current analyses often identify associations, but sophisticated bioinformatic pipelines and ML are needed to decipher the complex host-microbe-drug interactions and delineate true causality [98,102].

### Complexities of host-microbiome interactions

The relationship between the host and microbiome is profoundly bidirectional and complex. Host factors like genetics, immune status, and the disease itself significantly shape the microbiome, which in turn influences drug metabolism and treatment response [99,103,104]. This creates a challenge in isolating the microbiome's specific contribution from other host factors. A critical knowledge gap is the existence and role of intratumoral microbes within the unique tumor microenvironment (TME) of hematologic malignancies, which remains largely unexplored compared to solid tumors [98,99]. Furthermore, the microbiome's immunomodulatory effects, which can impact responses to therapies like immune checkpoint inhibitors (ICIs), are poorly understood in the context of blood cancers [99,103]. The added layer of treatment-related immunosuppression further alters these interactions in ways that affect efficacy and toxicity [52,99].

### Barriers to clinical translation

Translating microbiome-based interventions into hematologic practice requires careful attention to safety, manufacturing quality, and regulatory oversight. Because FMT, probiotics, defined consortia, and engineered strains involve living biological material, their clinical use demands rigorous donor screening, product characterization, potency testing, and post-intervention safety monitoring. For investigational uses, current regulatory pathways generally require appropriate IND oversight and compliance with CMC expectations for live biotherapeutic products [[105], [106], [107]]. Ethical issues are equally important in this immunocompromised population. Informed consent should clearly address the investigational nature of these therapies, uncertain long-term risks, and the possibility of limited benefit, while trial designs should include structured surveillance for infections and other adverse events [108,109].

### Novel therapeutic approaches and future directions

Against this backdrop, translational work is increasingly focused on integrating microbiome modulation with clinical care. Approaches such as FMT, probiotics, prebiotics, and dietary interventions are being evaluated as adjuncts to improve treatment response and reduce toxicity, while the field gradually moves toward more targeted, strain-specific strategies [103,110].

Synthetic biology offers exciting future directions by enabling the engineering of microbial strains with enhanced capabilities, such as producing anticancer agents or modulating specific immune responses directly within the TME [98,102]. However, ensuring the stability and safety of these engineered systems in immunocompromised patients remains a significant technical challenge [52,99]. The clinical pipeline is beginning to reflect these advances. Beyond FMT trials, several early-phase studies are evaluating defined consortia. For example, the SER-109 trial (a consortium of Firmicutes spores) demonstrated efficacy in preventing recurrent C. difficile infection and is being investigated for applications in oncology [111]. In hematology, trials are assessing oral supplements containing Lactobacillus and Bifidobacterium strains to reduce mucositis during induction chemotherapy for AML. These studies, though preliminary, are critical for establishing the safety framework and proof-of-concept needed to advance more complex engineered therapeutics.

Another exploratory frontier is the development of microbiome-informed decision-support models that integrate microbial features with clinical variables to support hypothesis-driven dosing and monitoring; however, these tools remain unvalidated and require prospective multicenter confirmation before clinical use [8,102]. Finally, the discovery and validation of microbial biomarkers (e.g., Akkermansia muciniphila for ICI response) could guide treatment selection and identify patients most likely to benefit from microbiome-modulating interventions [8,110]. Rigorous validation in independent cohorts and prospective trials is essential for this transition to clinical utility [8,102].

## Conclusion

Collectively, the evidence reviewed here indicates that the gut microbiome is an active modulator of drug metabolism, immune tone, and mucosal integrity in hematologic malignancies, shaping both therapeutic efficacy and toxicity across chemotherapy, immunotherapy, and transplantation. These observations support microbiome-based biomarkers and interventions as promising adjuncts for future precision hematology, but their clinical use will depend on reproducible validation, standardized methods, and clear safety and regulatory frameworks, particularly in immunocompromised patients.

Future research should therefore prioritize causal inference, prospective validation, and clinically actionable microbiome-guided strategies that can be integrated responsibly into hematologic care. Taken together, the pharmacomicrobiome represents an important but still emerging component of precision hematology, with substantial technical, regulatory, and validation hurdles remaining before routine clinical implementation.

**Acronyms and abbreviations**
16S rRNA: 16S ribosomal RNA
AML: Acute myeloid leukemia
BM: Bone marrow
CAR-T: Chimeric Antigen Receptor T-cell(s)
CRS: Cytokine Release Syndrome
CTLA-4: Cytotoxic T-lymphocyte-associated protein 4
C. difficile: Clostridioides difficile
EcN: E. coli Nissle
E. coli: Escherichia coli
EMA: European Medicines Agency
ESBL: Extended-spectrum beta-lactamase
FDA: U.S. Food and Drug Administration
FMT: Fecal Microbiota Transplantation
G-CSF: Granulocyte colony-stimulating factor
GM-CSF: Granulocyte-macrophage colony-stimulating factor
GF: Germ-free
GPCR(s): G-protein-coupled receptor(s)
GMP: Good Manufacturing Practice.
GVHD: Graft-versus-host disease.
HDAC: Histone deacetylase
HMA(s) / HMAs: Hypomethylating agent(s)
HSPC(s): Hematopoietic stem and progenitor cell(s)
HSCT: Hematopoietic stem cell transplantation
ICANS: Immune Effector Cell-Associated Neurotoxicity Syndrome
ICI(s) / ICIs: Immune Checkpoint Inhibitor(s)
IFN-γ: Interferon-gamma
IL-1, IL-6, IL-10: Interleukin-1, Interleukin-6, Interleukin-10
IMiDs: Immunomodulatory imide drugs
LBPs: Live Biotherapeutic Products
MDS: Myelodysplastic syndromes
ML: Machine learning
NGPs: Next-generation probiotics
PD-1 / PD-L1: Programmed cell death protein 1 / its ligand
SCFA / SCFAs: Short-chain fatty acid(s)
SHIME: Simulator of Human Intestinal Microbial Ecosystem
Treg(s): Regulatory T cell(s)
Th1, Th17: T helper 1 and T helper 17 cell subtypes.
TPMT: Thiopurine S-methyltransferase
TNF-α: Tumor necrosis factor alpha
VRE: Vancomycin-resistant Enterococcus.

## Funding

This research did not receive any financial support from public, commercial, or nonprofit organizations.

## Ethics approval and consent to participate

Not applicable.

## Consent for publication

Not applicable.

## CRediT authorship contribution statement

**Amr Ali Mohamed Abdelgawwad El-Sehrawy:** Writing – review & editing, Validation, Conceptualization. **Hamed Soleimani samarkhazan:** Writing – review & editing, Writing – original draft, Visualization, Validation, Conceptualization.

## Declaration of competing interest

The authors declare that they have no competing interests.

## Data Availability

No datasets were generated or analysed during the current study.
